# Pregnancy-associated stroke –a systematic review of subsequent pregnancies and maternal health

**DOI:** 10.1186/s12884-019-2339-y

**Published:** 2019-05-28

**Authors:** Liisa Karjalainen, Minna Tikkanen, Kirsi Rantanen, Hannele Laivuori, Mika Gissler, Petra Ijäs

**Affiliations:** 1 Department of Neurology, University of Helsinki and Helsinki University Hospital, Biomedicum Helsinki, P.O. Box 700, FI-00029 HUS Helsinki, Finland; 2 Department of Obstetrics and Gynaecology, University of Helsinki and Helsinki University Hospital, Helsinki, Finland; 3 Medical and Clinical Genetics, University of Helsinki and Helsinki University Hospital and Institute for Molecular Medicine Finland, Helsinki Institute of Life Science, University of Helsinki, Helsinki, Finland; 4 Department of Obstetrics and Gynaecology, Tampere University Hospital and Tampere University, Faculty of Medicine and Health Technology, Tampere, Finland; 5 Department of Information Services, National Institute of Health and Welfare, Helsinki, Finland; 6 Department of Neurobiology, Care Sciences and Society, Karolinska Institute, Stockholm, Sweden

**Keywords:** Stroke, Pregnancy, Postpartum period, Puerperium, Pregnancy-associated stroke, Follow-up, Recurrence, Subsequent pregnancies, Pregnancy outcome, Future health, Cardiovascular disease

## Abstract

**Background:**

Pregnancy-associated stroke is a rare but life-threatening event, with an estimated incidence of 30/100000 deliveries. Data on the risk of stroke recurrence and the risk of other adverse pregnancy outcomes are essential for adequate counselling and surveillance in subsequent pregnancies. The aim of this systematic review is to describe the implications of a pregnancy-associated stroke for the future health of these women.

**Methods:**

We searched Ovid Medline, PubMed, Cochrane Library and CINAHL for articles published in 1980–2018. Articles including women with pregnancy-associated stroke and information on at least one of the following outcomes were included: 1) recurrence of stroke during subsequent pregnancy, 2) number and course of subsequent pregnancies and their outcomes and 3) subsequent cardiovascular health.

**Results:**

Twelve articles were included in the review, with six providing information on subsequent pregnancies, four on subsequent maternal health and two on both. The included articles varied greatly in terms of study design, length of follow up and reported outcomes. We found 252 women with pregnancy-associated stroke for whom the outcomes of interest were reported: 135 women with information on subsequent pregnancies and 123 women with information on future health. In total, 55 pregnancies after stroke were found. In the majority of studies, the incidence of pregnancy complications was comparable to that of the general population. The risk of stroke recurrence during pregnancy was 2%. Data on subsequent health of these women were limited, and the quality of the data varied between the studies.

**Conclusions:**

Data on subsequent pregnancies and health of women with a history of pregnancy-associated stroke are limited. Further research on this topic is essential for adequate counselling and secondary prevention.

**Electronic supplementary material:**

The online version of this article (10.1186/s12884-019-2339-y) contains supplementary material, which is available to authorized users.

## Background

A stroke during pregnancy or puerperium is a rare event, but markedly affects the future life of the woman and her family. Pregnancy-associated stroke (PAS) accounts for up to 15% of maternal deaths [1]. The reported incidence of PAS and the distribution of stroke subtypes, ischaemic stroke (IS), intracerebral haemorrhage (ICH), subarachnoid haemorrhage (SAH) and cerebral venous thrombosis (CVT), varies greatly due to different inclusion criteria and study designs. A recent meta-analysis reported an incidence of 30 strokes per 100,000 deliveries among all pregnancies [2]. The incidence of PAS varies depending on gestational age, with the greatest risk observed during the third trimester, delivery and the early postpartum period [3, 4]. The latest studies indicate an increase in the PAS incidence, primarily as a result of an increase in hypertensive disorders of pregnancy and cardiac disease in pregnant women [5, 6].

Pregnancy and postpartum period increase the stroke risk compared to non-pregnant time. The estimated incidence of stroke during reproductive age is 10–20 per 100,000 person-years [7, 8] and during pregnant and puerperal time 34 per 100,000 person-years (assuming the duration of pregnancy to be 40 weeks and defining puerperium as 6 weeks postpartum). Underlying diseases such as prothrombotic conditions, heart disease and anomalies of cerebral vasculature are risk factors for stroke also during pregnancy [9–12], while women with PAS are less likely to have conventional risk factors than non-pregnant women who suffer a stroke at the same age [13, 14]. Pregnancy-related increase in stroke risk is thought to be a result of hemodynamic and coagulation system changes and pregnancy complications such as hypertensive disorders of pregnancy [5, 10, 12, 15–17], infections [9, 10, 12], haemorrhage [10] and fluid and electrolyte imbalance [10, 12].

The outcome of stroke varies, but full recovery is possible, particularly in high-income countries. Since these women are of reproductive age, they often wish to conceive again. However, there is significant uncertainty surrounding the potential risks related to subsequent pregnancies and data on future health and long-term prognosis of these women are limited. The aim of this systematic review is to summarise the current knowledge on the subsequent pregnancies and future health of women with PAS and knowledge gaps in order to highlight the need for further research on the topic, essential for adequate counselling, pregnancy surveillance and secondary prevention throughout life in the future.

## Methods

The literature search was conducted to identify studies reporting subsequent pregnancy outcomes and future cardiovascular health among women with a history of PAS in accordance with the Preferred Reporting Items for Systematic Reviews and Meta-Analysis (PRISMA) guidelines [18]. Relevant studies were identified covering the period from January 1980 to September 2018. We searched Ovid Medline, PubMed, Cochrane Library and CINAHL with the following terms: “stroke” or “cerebrovascular disorder” or specific stroke types separately and “pregnancy” or "postpartum period and “follow-up” or “recurrence” (see Additional file 1 for detailed search strategy). Reference lists from relevant articles were assessed in order to identify additional potential articles. Articles were included if they were published in English and included women with a history of pregnancy-associated or puerperal stroke. Of the stroke subtypes, we included IS, CVT, ICH and SAH. TIA was excluded. Articles were included if information on at least one of the following outcomes was provided: 1) recurrence of stroke during subsequent pregnancy, 2) number of subsequent pregnancies and their outcomes, 3) subsequent cardiovascular health. We excluded case reports and studies that limited their follow-up to the mortality and neurological recovery from the initial stroke.

Two authors (LK and PI) screened the titles and abstracts identified through the literature search. If eligibility remained unclear, the full article was reviewed. Disagreements were resolved through consensus. Data on the number of women with PAS, the number of women with PAS who had subsequent pregnancies, the number of pregnancies and their outcomes and complications, data on future health and the duration of the follow-up were extracted from the eligible articles.

## Results

**Fig. 1:**
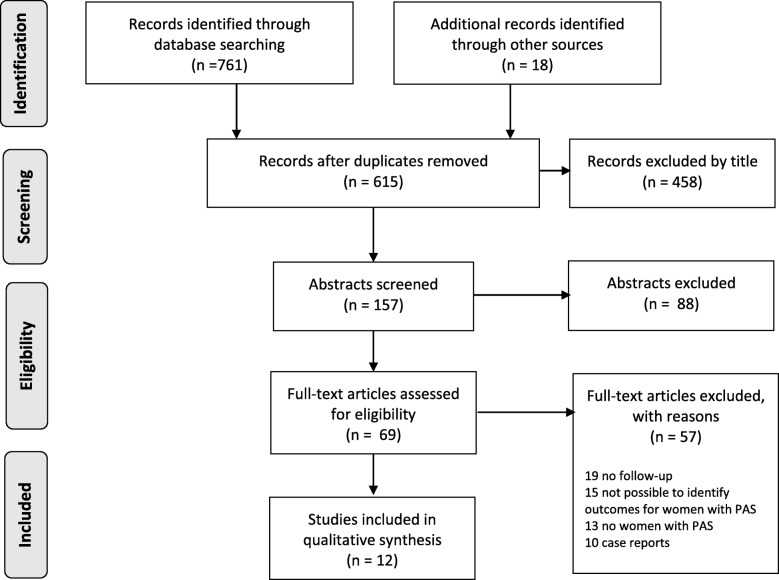
Flow diagram of the review process

Figure 1 shows the flow diagram of the review process. We screened 615 records, 157 abstracts and 69 full-texts articles. Fifteen articles included women with PAS but did not provide sufficient data to identify the outcomes for the subgroup. All twelve included articles mainly reported outcomes of women with a history of non-pregnancy associated stroke (NPAS); only 3–33% of women included in the cohorts had suffered a PAS. We found no studies focusing solely on women with PAS. Table 1 shows the details of the included studies [19–30]. We identified 252 cases with PAS who were included in the follow-up of the outcomes of interest: 135 women for subsequent pregnancies and 123 women for future health. CVT was the index event in 103/135 women (76%) for whom subsequent pregnancies were reported.

**Table 1:** Study details

Author	Publication year	Country	Data collection years	Study cohort	Method of follow-up	Women in cohort (n)	PAS women (n)	PAS women (%)	Type of PAS	Duration of follow-up	Subsequent pregnancies	Future health
Lanzino et al. [19]	1991	Italy (single centre)	1978–1988	prospective cohort of patients aged 16–45 with IS or TIA	clinical visit or telephone interview	59	2	3	IS (pp)	mean 5.8 y, range 6mo-11 y		x
Lamy et al. [20]	2000	France (9 centres)	1987–1997	retrospective cohort of women aged 15–40 admitted for first IS or CVT	written questionnaire, phone interview, patient records verification of substequent strokes	411	37	9	28 IS/9 CVT	mean 5, SD 2.4, range 0.8–11.3 y	x	
Mehraein et al. [21]	2003	Germany (single centre)	1976–1996	retrospective cohort of patients treated for CVT	telephone interview, mailed questionnaire	39	4*	10	CVT (2 pp., 1 w9, 1 w40)	mean 10.25, range 1–20 y	x	
Breteau et al. [22]	2003	France (2 centres)	1995–1998	retrospective cohort of patient with CVT from two hospitals follow up by visit/phone	visit or telephone interview	42	3	7	CVT (pp)	median 36, range 12–60 mo		x
Coppage et al. [21]	2004	USA (3 centres)	1990–2002	reprospective cohort of pregnant women with a history stroke	hospital charts	23	4	17	1 CVT (pp); 1 NS (w6); 1 NS (w39); 1 IS (pp)	nr	x	
Ferro et al. [23]	2004	multinational, multicentre	1998–2001	prospective international multicentre study of CVT patients	clinical visits, telephone interview	465	77	17	CVT (24 p, 53 pp)	mean 18.6, SD 11.1, median 16 mo	x	
Appenzeller et al. [24]	2005	Brazil (single centre)	1992–2002	retrospective cohort patients (subgroup of women) with CVT	follow up by neurology/hematolyogy department	18	6	33	CVT (pp)	mean 46, range 11–145 mo	x	x
Ertresvg et al. [30]	2007	Norway (single centre)	1997–1999	prospective case-control study of women referred to neurology due to transient neurological symptoms	mailed questionnaires	41	2	5	IS (p)	5 y		x
Crovetto et al. [25]	2012	Italy (single centre)	2000–2009	retrospective cohort of w12 beyond pregnant women with a history of IS	contact at least at 6 mo, phone contact in case of no show	24	1	4	IS (p)	nr	x	
Gastrich et al. [26]	2012	New Jersey (non federal hospitals)	1994–2009	retrospective case-control register study of pre-eclamptic women with or without MI/stroke	register/hospital charts	353	90	25	SAH/ICH/IS/CVT***	up to 16 years		x
Ciron et al. [27]	2013	France (4 centres)	1995–2012	retrospective cohort of women with CVT at age 15–40	telephone interview, clinical visit offered	62	6	10	CVT (4 p, 2 pp)	mean 89.5+/−60.6 mo, median 76 mo, ~ 6 y	x	
Alebeek et al. [28]	2018	Netherlands (single centre)	1980–2010	prospective cohort of patients aged 18–50 with IS or TIA	telephone interview, mailed questionnaire	213	20**	9	IS	mean 14.8, SD 10.0 y	x	x

*PAS* Pregnancy-associated stroke, *p* pregnancy, *pp* postpartum, *w* pregnancy week, *IS* ischemic stroke, *ICH* intracerebral hemorrhage, *SAH* subarachnoid hemorrhage, *CVT* cerebral venous thrombosis, *y* year, *mo* month, *nr* not reported. *It is possible that there were more womenwith PAS in the cohort, but it was stated that 4 PAS women had subsequent pregnancies. ** Study included patients with ischemic stroke (*n* = 9) or TIA (*n* = 11) as PAS types, but did not specify outcomes between these subtypes. *** Numbers not specified

### Course of subsequent pregnancies

Of the 52 women with a history of PAS, 26 (50%) had subsequent pregnancies; the studies by Ciron et al. [27] and Ferro et al. [23] were excluded from this calculation, as they do not report the number of women with subsequent pregnancies. The included studies reported a total of 55 pregnancies. Outcomes were reported in 42 of these pregnancies and they were the following: 32 live births (76%), one foetal death (2%), six miscarriages (14%) and three induced abortions (7%). Pregnancy complications were reported by all but one study [20]. The studies included 22 pregnancies that proceeded beyond 22 gestational weeks, i.e. excluding miscarriages and induced abortions. Of these 22 pregnancies, 17 (77%) were uncomplicated/uneventful, three (13%) were complicated by premature rupture of membranes (< 37 gestational weeks), one (5%) with pre-eclampsia and one (5%) with foetal death. All of the complications reported were from the study by Coppage et al. [21]. The information on subsequent pregnancies and recurrence of stroke during pregnancy is presented in Table 2.

**Table 2:** Subsequent pregnancies and their outcomes in women with a history of PAS

Author	PAS women (n)	CVT (n)	IS (n)	NS (n)	Women with subsequent pregnancies (n)	Pregnancies (n)	Miscarriage (n)	Induced abortion (n)	Live births (n)	Fetal death (n)	Pregnancy > w22 complications raported (n)	Uncomplicated pregnancies (n)	PROM (n)	Pre-eclampsia (n)	Stroke recurrence(n)	Underlying disease
Appenzeller et al. [24]	6	6	0	0	2	2	nr	nr	2	0	2	2	0	0	0	none
Lamy et al. [20]	37	9	28	0	15	24	nr	nr	11	0	nr	nr	nr	nr	nr	2 CVT patients: hematologic disease
Crovetto et al. [25]	1	0	1	0	1	1	0	0	1	0	1	1	0	0	0	Antiphospholipid syndrome
Coppage et al. [21]	4	1	1	2	4	9	3	0	5	1	6	1	3	1	0	1 patient PSD; 1 patient PCD; 2 patients uncontrolled hyprtension
Ciron et al.* [27]	6	6	0	0	nr	2	1	1	0	0	0	0	0	0	1	1 patient: sickle cell disease
Mehraein et al. [29]	4	4	0	0	4	6	1	0	5	0	5	5	0	0	0	nr
Ferro et al. ** [23]	77	77	0	0	nr	11	1	2	8	0	8	8	0	0	0	nr
Total	135	103	30	2	26	55	6	3	32	1	22	17	3	1	1	

*CVT* Cerebral vein thrombosis, *IS* Ischemic stroke, *NS* stroke type not specified, *PROM* premature rupture of membranes, *PSD* Protein S deficiency, *PCD* Protein C deficiency, *nr* not reported for women with PAS. *Ciron et al. reported that in 9.7% (*n* = 6) of 62 women with CVT in reproductive age it was related to pregnancy. The authors report the number of pregnancies and their outcomes for one women with a PAS history, but do not state whether the other women with PAS history had pregnancies. **Ferro et al. reported that there were 8 uncomplicated pregnancies, 1 miscarriage and 2 induced abortions, but do not state amongst how many women these pregnancies occurred

Alebeek et al. [28] assessed the occurrence of pregnancy complications in women with a history of stroke (IS or TIA) using the Dutch general population as a control group. The data for the women with a stroke history were self-reported and collected by telephone interview and the data to represent the general population was retrieved from the Dutch Perinatal Registry or Dutch HELLP foundation. The patients and controls were not matched on common risk factors for stroke and pregnancy complications (smoking, thrombophilia, hypertension, hypertensive disorders of pregnancy). In the subgroup of 20 women with PAS, 50% had experienced more than one miscarriage, 5% more than one foetal death, 25% gestational hypertension, 20% pre-eclampsia, 20% HELLP and 10% gestational diabetes. Substantially lower rates for the Dutch general population were reported: 13.5, 0.9, 11.7, 0.5, 0.5 and 1.8%, respectively. The study did not report the timing of the pregnancy outcomes in relation to the PAS, i.e. whether the complications occurred during the same pregnancy or not.

### PAS recurrence

In the 55 pregnancies among women with a history of PAS, one (2%) recurrent PAS occurred. This was a first trimester CVT in a woman with a history of CVT during pregnancy, sickle cell disease as an underlying condition. She used no prophylactic anticoagulant medication during the pregnancies following the primary CVT. The pregnancy was terminated. The study of Ciron et al. [27] did not provide details on the primary or the recurrent CVT (i.e. the location or extend of CVT or did CVT result in ischemic or haemorrhagic stroke). In addition, four articles reporting subsequent health of women after PAS [19, 22, 28, 30] stated there were no recurrent PAS.

### Subsequent health

Table 3 presents information on the subsequent health of women with PAS. The articles varied greatly in terms of the diseases, outcomes included in follow-up and outcomes reported for women with PAS. Often all outcomes of interest were not specifically reported for women with PAS. The follow-up time varied from 6 months to 16 years after the index stroke. The initial type of stroke was not specified in 90/123 women (73%) whose subsequent health was assessed and reported. During the follow-up there were altogether three vascular events, all ISs or TIAs from the study of Alebeek et al. [28]. The studies reporting long-term mortality included 101 women and 10 (10%) of these women died during the follow-up time up to 16 years, four from cardiovascular causes and six from other causes. All of these women were from the same cohort from Gastrich et al. [26] and had pre-eclampsia in association with the index stroke.

**Table 3:** Future health of women with PAS

Author	Women in cohort (n)	PAS women *n* (%)	Types of events included in follow-up	Cardiovascular events reported for PAS	Mortality	Underlying condition	Duration of follow-up
Alebeek et al. (2018) [28]	213	20 (9)	Stroke or other arterial event, eg. MI or cardiovascular procedure	3 recurrent ischaemic strokes	nr	2 SLE, 2 APS, 7 hypertension, 1 DM, 6 smoking	mean 14.8, SD 10.0 y
Appenzeller et al. (2005) [24]	18	6 (33)	CVT or other thrombotic event	none	none	none	mean 46, range 11–145 mo
Breteau et al. (2003) [21]	42	3 (7)	CVT recurrence, DVT, PE, any other health problem leading to hospitalisation	no recurrent CVT	none	nr	median 36, range 12–60 mo
Ertresvg et al. (2007)	41	2 (5)	Any new diseases	none	none	1 APS + pre-eclampsia, 1 smoking + family histrory of stroke + FV Leiden heterozygous	5 y
Gastrich et al. (2012) [26]	353	90 (25)	MI, stroke, cardiovascular death, death from any cause	no recurrent stroke, no MI	4 cardiovascular deaths, 6 other deaths	pre-eclampsia	up to 16 years
Lanzino et al. (1991) [19]	59	2 (3)	Cerebral ischemia, MI, death	no recurrent strokes	nr	nr	mean 5.8 y, range 6 mo-11 y

*CVT* cerebral venous thrombosis, *MI* myocardial infarction, *PE* pulmonary embolism, *DVT* deep venous thrombosis, *SLE* systemic lupus erythematosus, *APS* antiphospholipid syndrome, *DM* diabetes mellitus, *y* year, *mo* month, *nr* not reported for women with PAS

## Discussion

During the past two decades knowledge on the incidence and risk factors of PAS has accumulated. By contrast, data on subsequent pregnancies and health of women with PAS are scattered, incompletely reported and limited information is available. The systematic review suggests that the incidence of pregnancy complications is comparable to that reported for women with a history of stroke (related or not related to pregnancy) [20, 21, 27, 31]. Miscarriage seems to be as common among women with PAS as in the general population [32], but induced abortion is slightly more infrequent than in the general population [33]. This may reflect more careful family planning after PAS. One study reported less favourable obstetric prognosis, more severe pregnancy complications and more miscarriages and foetal deaths in women with a history of stroke than in the general population [28]. However, there are several methodological problems in the study. Most importantly, the ascertainment and data collection differed between the women with PAS and controls and they were not matched on risk factors for adverse pregnancy outcomes, such as smoking, hypertension and prothrombotic conditions, which all are also risk factors for stroke. Therefore, the findings may result from confounding risk factors. Also the frequencies of adverse pregnancy outcomes reported in the Dutch Perinatal Registry for the general populations are low and possibly under-reported [28], which may overestimate the differences between women with a stroke history and general population.

Based on limited number of published cases, the proportion of women with subsequent pregnancies after PAS was relatively high, 50%. It is noteworthy that the percentage is based on less than half of the women with PAS included in the analysis of subsequent pregnancies, since two studies [23, 27] did not provide the data on number of women with subsequent pregnancies. This may overestimate the pregnancy rate. In previous studies, the proportion of young women with a history of stroke (related or not related to pregnancy) and subsequent pregnancies has ranged between 26 and 40% [20, 27, 29, 31, 34]. Lamy et al. [20] found no difference in the number of subsequent pregnancies between women with a history of IS or CVT and age- and parity-matched controls from the general population. However, one-third of women with a history of stroke indicated that they would have wished for more pregnancies after their stroke and the most common causes for hesitation were concerns of recurrent stroke, medical advice against a new pregnancy and residual disability [20].

We found one recurrent PAS, CVT associated to sickle cell disease. Even though the number of patients is very limited, the recurrence of stroke during pregnancy or puerperium seems to be rare. The same applies to young stroke patients whose initial strokes have not been pregnancy related. The recurrence is mainly limited to women with underlying haematological diseases (such as protein C and S deficiency, sickle cell disease, antiphospholipid syndrome or systemic lupus erythematosus), cerebrovascular disease (arteriovenous malformations, aneurysms or Moyamoya disease) or maternal cardiac disease [20, 21, 31, 35, 36]. This is exemplified by a study by Fischer-Betz et al. [35] who reported pregnancy outcomes and recurrence of stroke during pregnancy for 20 women with antiphospholipid syndrome and a history of IS or TIA. The possible temporal connection of index stroke to pregnancy was not reported. Among subsequent pregnancies, stroke recurred in 15%, while preeclampsia occurred in 34% of pregnancies and 8.7% of pregnancies resulted in neonatal death. Further, Soriano et al. [36] reported similar results in a study of thromboembolic complications of pregnancy and pregnancy outcomes in 12 women (15 pregnancies) with a history of stroke (IS, TIA and amaurosis fugax) and an underlying thrombophilic disorder who used LMWH and aspirin 100 mg until 6 weeks’ postpartum. Five of these 12 women (42%) in the cohort had a history of PAS. Four patients (33%) in this cohort had recurrent thromboembolic events (1 TIA, 1 amaurosis fugax in two subsequent pregnancies, 1 paresthesia of the left arm, 1 deep venous thrombosis and pulmonary embolism 3 weeks postpartum after discontinuing the preventive medication contrary to advice). Pre-eclampsia complicated five pregnancies (33% of all), leading to preterm delivery in three pregnancies (20%), of which one at 27 gestational weeks, resulting in neonatal death (7% of newborns). Even though the proportion of PAS in this cohort was only 42%, this may indicate that also PAS women with an underlying predisposing condition are at greater risk of recurrent PAS, other thrombotic events and adverse pregnancy outcomes than women whose index PAS has been cryptogenic.

The studies reporting subsequent health of women after PAS differed markedly in terms of settings and outcomes included in the follow-up. In 73% of the women whose future health was reported, the stroke subtype was not specified. During the follow-up ranging from 6 months to 16 years, altogether three ISs occurred among 123 women, i.e. 2% of the women suffered a NPAS. All of these women were from the same cohort of 20 women with PAS, the cumulative 20-year risk being 15.6% when assessing this cohort separately. Hindfelt et al. [37] found 11% of young IS survivors suffered a recurrence during the 13- to 26-year follow-up. The difference in their results compared with ours may be explained by a shorter follow-up time in the studies of our review, but also by a lower prevalence of traditional stroke risk factors among PAS women. Regarding mortality, we found a cumulative mortality rate of 10% after a follow-up ranging from 6 months to 16 years. This is in line with previous studies on the long-term prognosis of young patients with IS that have reported a cumulative 2- to 10-year mortality of 10% [38, 39].

This study has a number of limitations. All studies included in this review were comprised of mainly women with a history of stroke that was not associated with pregnancy, the proportion of PAS varying from 3 to 33%. Analyses were performed and results presented for the entire cohort instead of separating patients by the timing of the stroke, making it impossible to extract all outcomes included in the study for women with PAS specifically. The information about women with PAS, their subsequent pregnancies and their outcomes often had to be retrieved from tables in the articles or even from the discussion. A good example is the total number of pregnancies reported in the studies; two studies [20, 24] did not report miscarriages or induced abortions separately for women with PAS and Ciron et al. [27] reported outcomes of pregnancies for only one woman with PAS, but did not state whether other women with PAS had pregnancies and what their possible outcomes were. Therefore, the total number of pregnancies for women with PAS in the included studies may be more than 55. Lamy at al. [20] did not report pregnancy complications, which may underestimate their incidence. The quality of information varied and was especially scant regarding future cardiovascular health. The length of the follow-up period was insufficient for evaluation of long-term cardiovascular outcome and mortality. These gaps in reporting reflect that women with PAS were not the main focus in these articles. Altogether, the limited number of cases and incomplete reporting causes uncertainty for the interpretation of result. However, the results revealed an evident gap in knowledge that warrants research on this topic, preferably in a population-based setting and with a sufficiently long follow-up.

## Conclusion

PAS is a rare event with potentially devastating consequences for the everyday life of the young family. In the systematic review of subsequent pregnancies and health of these women, we found the data to be limited. In the context of published literature, half of women with a history of PAS have subsequent pregnancies, their outcomes are generally good and the stroke recurrence rate during subsequent pregnancies is low. This is reassuring when it comes to counselling women with a desire to conceive. An increased risk of adverse events and recurrence of PAS may be related to subsequent pregnancies of women with underlying diseases such as prothrombotic conditions, anomalies of cerebral vasculature and chronic heart disease. According to the results of this review, the prognosis of future health is similar for women with a history of PAS and patients with a history of stroke at young age, unrelated to pregnancy. However, further research on this topic is needed to improve pregnancy surveillance, preventive medication in subsequent pregnancies and secondary prevention throughout life.

## Additional file

Additional file 1: Detailed search strategy for Ovid Medline, PubMed, CINHAL and Cochrane Library. (PDF 9 kb)

## Abbreviations

CVT: Cerebral venous thrombosis
HELLP: Haemolysis, elevated liver enzyme, low platelet count
ICH: Intracerebral haemorrhage
IS: Ischaemic stroke
LMWH: Low molecular weight heparin
PAS: Pregnancy-associated stroke
SAH: Subarachnoid haemorrhage
SLE: Systemic lupus erythematosus
TIA: Transient ischaemic attack
