# Supplementary material for: Pregnancy-associated stroke –a systematic review of subsequent pregnancies and maternal health
Source: BMC Pregnancy Childbirth. 2019 May 28;19:187. doi: 10.1186/s12884-019-2339-y (PMC6540366; doi:10.1186/s12884-019-2339-y)
Supplement: Supplementary file 1 — Detailed search strategy for Ovid Medline, PubMed, CINHAL and Cochrane Library. (PDF 9 kb) [file 12884_2019_2339_MOESM1_ESM.pdf]

Additional File 1: Detailed search strategy

PubMed and Ovid Medline were searched in early September and CINAHL and Cochrane Library in early October 2018 with the following strategies:

PubMed

Search **((((((((((((((((((((((((((("stroke") OR "cerebrovascular disorders") OR "intracerebral hemorrhage") OR "subarachnoid hemorrhage"))) OR "ischemic stroke") OR "cerebral venous thrombosis") OR "intracranial thrombosis") OR "sagital sinus thrombosis"))) OR ("intracranial embolism and thrombosis")) OR "transient ischemic attack"))) AND (((((("pregnancy")) OR "pregnancy complications") OR "puerperal disorders") OR "postpartum period") OR "pregnancy complications cardiovascular")) AND ((("follow up studies") OR "recurrence") OR "pregnancy outcome")) AND "adult")) AND "english"[Language]** Sort by: **Title**

The search yielded 268 articles.

Ovid Medline

1 exp STROKE/ or exp STROKE, LACUNAR/ (116819)

2 exp Cerebrovascular Disorders/ (337018)

3 exp Cerebral Hemorrhage/ (31876)

4 exp Subarachnoid Hemorrhage/ (19779)

5 exp Intracranial Thrombosis/ or exp Sinus Thrombosis, Intracranial/ or exp "Intracranial Embolism and Thrombosis"/ or exp Cerebral Veins/ (23441)

6 exp Ischemic Attack, Transient/ (19516)

7 1 or 2 or 3 or 4 or 5 or 6 (338872)

8 Pregnancy/ (826941)

9 exp Postpartum Period/ (59385)

10 exp Pregnancy Complications/ (400048)

11 exp Puerperal Disorders/ (31575)

12 exp Pregnancy Complications, Cardiovascular/ (16596)

13 8 or 9 or 10 or 11 or 12 (875082)

14 exp Follow-Up Studies/ (599539)

15 exp RECURRENCE/ (172247)

16 exp Pregnancy Outcome/ (51470)

17 14 or 15 or 16 (789904)

18 7 and 13 and 17 (683)

19 limit 18 to (english language and humans and yr="1980 -Current") (580)

20 exp ADULT/ (6648550)

21 19 and 20 (344)

CINAHL

Query Limiters/Expanders Last Run Via Results

S4 S1 AND S2 AND S3 Search modes - Boolean/Phrase Interface - EBSCOhost Research Databases

Search Screen - Advanced Search

Database - CINAHL 122

S3 follow up study OR recurrence OR pregnancy outcomes Search modes - Boolean/Phrase Interface - EBSCOhost Research Databases

Search Screen - Advanced Search

Database - CINAHL 69,870

S2 pregnancy OR postpartum period OR pregnancy complications OR puerperium Search modes - Boolean/Phrase Interface - EBSCOhost Research Databases

Search Screen - Advanced Search

Database - CINAHL 128,359

S1 stroke OR cerebrovascular disorders OR intracerebral hemorrhage OR ischemic stroke OR transient ischemic attack OR cerebral venous thrombosis OR cerebral venous sinus thrombosis OR subarachnoid hemorrhage Search modes - Boolean/Phrase Interface - EBSCOhost Research Databases

Search Screen - Advanced Search

Database - CINAHL 74,920

Cochrane Library

ID	Search	Hits
#8	MeSH descriptor: [Pregnancy] explode all trees	6926
#9	MeSH descriptor: [Postpartum Period] explode all trees	1414
#10	MeSH descriptor: [Pregnancy Complications] explode all trees	9894
#11	MeSH descriptor: [Puerperal Disorders] explode all trees	1421
#12	MeSH descriptor: [Pregnancy Complications, Cardiovascular] explode all trees	322
#13	#8 or #9 or #10 or #11 or #12	14749
#14	MeSH descriptor: [Follow-Up Studies] explode all trees	55464
#15	MeSH descriptor: [Recurrence] explode all trees	11246
#16	MeSH descriptor: [Pregnancy Outcome] explode all trees	2992
#17	#14 or #15 or #16	66483
#23	MeSH descriptor: [Stroke] explode all trees	7761
#25	MeSH descriptor: [Cerebral Hemorrhage] explode all trees	885
#26	MeSH descriptor: [Subarachnoid Hemorrhage] explode all trees	520
#27	MeSH descriptor: [Ischemic Attack, Transient] explode all trees	648
#28	MeSH descriptor: [Intracranial Thrombosis] explode all trees	47
#29	MeSH descriptor: [Cerebrovascular Disorders] explode all trees	12696
#30	#23 or #25 or #26 or #27 or #28 or #29	12696
#31	#30 and #13 and #17	27
